# Interpretable multimodal reasoning for robo-advisory: the FinErva framework

**DOI:** 10.3389/frai.2025.1752580

**Published:** 2026-01-21

**Authors:** Jiarui Chi

**Affiliations:** PBC School of Finance, Tsinghua University, Beijing, China

**Keywords:** chain-of-thought, explainable artificial intelligence, investment decision support, lightweight and low cost, multimodal financial reasoning, robo-advisory

## Abstract

The rapid development of robo-advisory and quantitative investment has been accompanied by persistent concerns about limited personalization and the opacity of black-box models operating on multimodal financial information. This paper addresses these issues from a decision-support perspective by constructing FinErva, a multimodal chain-of-thought dataset tailored to financial applications. FinErva comprises 7,544 manually verified question–answer pairs, divided into two economically relevant tasks: contract and disclosure understanding (FinErva-Pact) and candlestick-chart-based technical analysis (FinErva-Price). Building on this dataset, the paper propose a two-stage training framework: Supervised-CoT Learning followed by Self-CoT Refinement, and apply it to eight vision–language models, each with fewer than 0.8 billion parameters. Empirical results show that those lightweight models approach the performance of finance professionals and clearly outperform non-expert investors. Overall, the findings indicate that appropriately designed multimodal chain of thought supervision enables interpretable modeling of key research tasks such as contract review and chart interpretation under realistic computational and deployment constraints, providing new data and methodology for the development of personalized, explainable, and operationally feasible AI systems in investment advisory and risk management.

## 1 Introduction

In recent years, robo-advisors have gradually emerged as a central form of retail wealth management (Eskandarany, 2024; Theodorakopoulos et al., 2025) and are widely regarded as a key technology for replacing human financial advisors (Jadhav and Mirza, 2025) with quantitative models, reducing service costs, and improving advisory efficiency (Sutiene et al., 2024). However, in practice there remains a substantial gap between robo-advisors and experienced human analysts in terms of personalization capabilities and decision quality in complex scenarios (Jadhav and Mirza, 2025). Existing studies and industry reports (Verma et al., 2025; Jung et al., 2018; Goswami et al., 2025) indicate that most current robo-advisors still rely on low-dimensional risk-preference questionnaires and pre-specified model portfolios (Boreiko and Massarotti, 2020), which are insufficient to capture investors' heterogeneous preferences and behavioral characteristics. As a result, the recommended portfolios tend to exhibit a “one-size-fits-all” pattern, and their decision performance often falls short of that of seasoned professional analysts (D'Acunto et al., 2019). This perception of “insufficient personalization and questionable decision quality” is one of the fundamental reasons why investors remain cautious about adopting robo-advisory services (Verma et al., 2025).

From an institutional perspective, achieving true personalization and specialization in stock investment decision-making and trading strategy research often requires the construction of proprietary models tailored to specific markets, asset classes, or even investment styles. These proprietary models not only demand substantial feature engineering and parameter tuning during the research and development phase, but also face a series of cost constraints (Cottier et al., 2025; Maple et al., 2024; Interpress, 2024), such as computational power, storage, latency control, and regulatory scrutiny, during the deployment phase (Paleyes et al., 2022; Sen et al., 2021). As the scale of models rapidly expands, the parameter size of large-scale pre-trained models has progressed from tens of billions to hundreds of billions, leading to a sharp increase in the computational power and data required for training and fine-tuning. This has made it increasingly difficult in practice to customize large models for a single institution or a single strategy. Even within relatively traditional machine learning frameworks, the introduction of methods such as ensemble learning and deep networks significantly raises the costs of model development and maintenance, not to mention the added complexity of incorporating language models and multimodal models on top of these approaches.

On the quantitative modeling side, most decision and regression frameworks used in investment management are still grounded in linear or generalized linear models (Kwon, 2025; Liu and Song, 2025; Feng et al., 2025; Tan et al., 2025) from classical factor models to regularized regressions and generalized linear risk models. While these approaches have delivered tractable estimation procedures, they are structurally limited in capturing the complex, nonlinear interactions and regime-dependent patterns that characterize modern financial markets. Empirical asset-pricing research (Bagnara, 2024; Chen et al., 2024) demonstrate that nonlinear machine learning models, such as tree ensembles and deep neural networks, are able to extract economically meaningful signals from high-dimensional characteristics and often generate substantial improvements in out-of-sample Sharpe ratios compared to leading linear benchmarks. Building upon this, Large Language Models (LLMs) take it a step further by extending the “high-dimensional pattern recognition” ability to unstructured text and even multimodal (text modal and vision modal) data. Through pre-training and transfer learning on large-scale corpora within the financial context, LLMs achieve effective optimization on highly non-convex objective functions. Even when faced with extremely high-dimensional decision spaces, they exhibit superior expressiveness and robustness compared to traditional linear frameworks.

However, simply relying on stronger pattern recognition abilities is not enough to drive the large-scale deployment of robo-advisors in real-world financial scenarios. For high-risk, heavily regulated financial businesses, the model's interpretability and auditability are just as important as predictive accuracy (Maier et al., 2022; Bussmann et al., 2020; Fritz-Morgenthal et al., 2022). On one hand, regulatory bodies and compliance departments need to trace and hold the model's decision-making logic accountable. On the other hand, end investors are more likely to entrust real wealth to automated systems if they understand how the model arrived at this asset allocation or trading recommendation. In the frontier of large model research (firstly introduced by Wei et al., 2023), the Chain-of-Thought (CoT) prompt has been proposed as a paradigm that allows the model to explicitly display its intermediate reasoning process: by guiding the model to generate step-by-step reasoning before providing the final conclusion, CoT not only significantly improves accuracy on complex reasoning tasks but also provides a direct entry point for human review and intervention in model decisions. Subsequent research (Zhang et al., 2024) has extended CoT to multimodal scenarios, showing that when jointly processing text and images, having the model output a structured reasoning chain can effectively align visual evidence with linguistic reasoning, thus enhancing performance and interpretability in multimodal question-answering and decision-making tasks. For robo-advisors, an interpretable Chain-of-Thought means that the model not only provides a “buy/sell/hold” conclusion but also clearly points to the underlying price trends, technical patterns, financial indicators, and textual information, thereby enhancing both decision accuracy and investor trust.

At the same time, real-world investment decisions are inherently multimodal. A human analyst typically integrates structured market and fundamental data (time series, ratios, and factor exposures), unstructured textual information (earnings calls, news, analyst reports), and visual signals (candlestick charts, technical indicators, and even screenshots of trading interfaces). However, to the best of our knowledge, research on “multimodal + Chain-of-Thought + large model” systems specifically tailored for investment decision-making scenarios remains scarce. Existing work (shown in Table 1) either lacks clear intermediate reasoning annotations or focuses solely on a single modality or task, making it difficult to comprehensively support the multimodal decision-making process modeling required for intelligent robo-advisors.

**Table 1:** Comparison of existing finance datasets: ✗* means text-extracted-format data, ✓* means partial correct.

**Dataset**	**Domain**	**Sample in Fin**	**MultiModal**	**Data description**	**CoT**	**GT**	**Language**	**Reasoning only from image**	**Image complexity**
Ant_Finance (Team A., 2023)	Fin	13K	✗	Understanding and reasoning	✗	✓*	ZH	✗	0
FinanceIQ (Team D. D., 2023)	Fin	Large enough	✗	Open-domain question	✗	✗	ZH	✗	0
FinQA (Chen et al., 2022a)	Fin	8.3K	✗*	Understanding and reasoning	✗	✓	EN	✗	0
Finance-Instruct (Flowers, 2025)	Fin	500K	✗	Reasoning, sentiment analysis	✗	✓*	Mul-lang	✗	0
BBF-Fin (Lu et al., 2023)	Fin	Large enough	✗	Understanding and generation	✗	✗	ZH	✗	0
MME-Finance (Gan et al., 2024)	Fin	1.2K	✓	Open-ended question	✗	✗	ZH,EN	✗	3
ConvFinQA (Chen et al., 2022b)	Fin	3.9K	✗*	Table understanding	✗	✓	EN	✗	0
TAT-QA (Zhu et al., 2021)	Fin	16.5K	✗*	Numerical reasoning	✗	✓*	EN	✗	0
FAMMA (Xue et al., 2025)	Fin	1.9K	✓	Understanding and reasoning	✗	✓	EN,FR	✓	2
Fin-Fact (Rangapur et al., 2024)	Fin	3.6K	✓	Fact judgment	✗	✓	EN	✗	1
PDF-VQA (Ding et al., 2023)	Fin	140K	✓	PDF understanding	✗	✓*	EN	✓	3
Sujet-finance-QA (Sujet and Allaa Boutaleb, 2024)	Fin	100K	✓	Understanding and generation	✗	✓	EN	✗	4,5
FinVis-GPT (Wang et al., 2023)	Fin	1M	✓	Open-ended question	✗	✗	ZH,EN	✗	3
FinErva (this work)	Fin	7.54K	✓	Understanding and reasoning	✓	✓	EN	✓	3,4,5

Image complexity from zero to five respectively indicates: no image, simple caption, structured tables, articles, or candlestick charts, images with noise such as handwriting and scan artifacts, highly noisy and semantically complex images that are difficult for humans to interpret. A red cross indicates that the criteria are not met, while a green checkmark indicates that the criteria are met.

Addressing the aforementioned research gap, this paper proposes and develops the FinErva (FINancial-llm-with-minERVA^1^-wisdom) framework, which aims to provide a systematic data foundation and a lightweight model solution for multimodal Chain-of-Thought research in the field of intelligent robo-advisors. Specifically, FinErva integrates three representative financial scenarios—financial contract and document understanding, real-world financial image interpretation, and technical analysis based on candlestick charts—to create the first multimodal Chain-of-Thought question-answer dataset for the financial domain. Each sample contains real financial images, carefully designed question-answer pairs, multiple-choice options with distractors, and manually verified step-by-step reasoning chains. Based on this dataset, this paper further proposes a lightweight fine-tuning pipeline: by incorporating vision feature extraction modules at the visual encoding layer to address the visual encoding issues of lightweight models, and performing two-stage CoT fine-tuning on a series of open-source vision-language models with parameter sizes under 0.8B. The first stage involves supervised Chain-of-Thought learning (Supervised-CoT-Learning), while the second stage focuses on model self-refinement (Self-CoT-Learning), thus enabling multimodal reasoning and interpretability for financial tasks while keeping deployment costs under control. The key contributions of this work are as follows:

- The first multimodal Chain-of-Thought dataset and task setting specifically designed for the financial domain: The FinErva system systematically covers key scenarios such as financial contract understanding, complex financial scene image analysis, and technical analysis of candlestick chart patterns. By characterizing multimodal question-answering and reasoning requirements within a unified framework, FinErva provides a high-quality data foundation for subsequent research on intelligent robo-advisors and large financial models.
- A reproducible and scalable low-cost, lightweight financial multimodal CoT fine-tuning pipeline: High-performance financial reasoning capabilities are achieved on vision-language models with parameter sizes under 0.8B. Experimental results show that the model fine-tuned on FinErva significantly outperforms both zero-shot in terms of accuracy. Additionally, the performance metrics approach or even exceed those of human experts with professional backgrounds (Table 2).
- From the perspective of “interpretable intelligent robo-advisors,” this paper organically integrates CoT, LLMs, and multimodal financial scenarios: Through explicit Chain-of-Thought outputs and a low-cost deployment solution, FinErva provides a feasible pathway for constructing the next generation of robo-advisory systems that are both interpretable and capable of multimodal perception. It also lays the methodological foundation for auditable AI in application scenarios such as financial regulation, compliance review, and risk management.

**Table 2:** Human evaluation accuracy.

**Human evaluation**	**Acc-pact**	**Acc-price**
Finance expert 1	72%	88%
Finance expert 2	64%	82%
Random participant 1	20%	56%
Random participant 2	32%	62%
**Fine-tuned model (ours)**	**68.29%**	**86.03%**

## 2 Related work

Large language models and generative AI have recently emerged as flexible building blocks for financial analytics, including portfolio optimization, risk management, algorithmic trading, robo-advisory, and ESG analytics. Studies consistently report that deep learning and LLM-based systems can improve predictive performance or reduce information-processing costs, but also that they introduce new challenges in terms of explainability, fairness, operational risk, and infrastructure investment. This section primarily discusses the current state of research in the various fields relevant to this study, and Sections 2.4, 2.5 respectively outline the technical value and financial significance.

### 2.1 Financial QA and document/table understanding

Studies focuse on financial QA and document or table understanding, where models are trained to answer questions about financial reports, prospectuses, or numerical tables. FinQA (Chen et al., 2022a) and ConvFinQA (Chen et al., 2022b) center on numerical reasoning over financial tables and textual contexts, simulating analyst-style questions based on financial documents. TAT-QA (Zhu et al., 2021) extends this paradigm to more complex table–text interactions, where answers require multi-step aggregation and cross-referencing between text and semi-structured tables. FinanceIQ (Team D. D., 2023) and Finance-Instruct (Flowers, 2025) provide large-scale instruction-style QA corpora for financial knowledge and task-oriented dialogue, while BBF-Fin (Lu et al., 2023) targets Chinese-language financial understanding and generation. Collectively, these benchmarks have enabled substantial progress in text-based and table-based financial reasoning. On the benchmarking side, FinBen (Xie et al., 2024) is proposed as a holistic financial benchmark for LLMs, covering a wide range of tasks including factual knowledge, numerical reasoning, and document understanding in finance. Complementary work such as Fino1 (Qian et al., 2025) and Fin-R1 (Liu et al., 2025) explores how reasoning-enhanced LLMs and reinforcement-learning-based alignment can improve financial question answering and reasoning quality on text-only financial tasks.

However, most of these studies share three structural limitations. First, they operate primarily on structured or semi-structured inputs (tables and machine-readable text) and thus under-represent realistic financial artifacts such as scanned contracts, handwritten annotations, or chart screenshots. Second, they partially provide final answers (Ground Truth), but not explicit, human-authored reasoning trajectories that could be used to train or evaluate chain-of-thought explanations. Third, the target tasks are usually framed as isolated QA problems rather than as components of a broader, multimodal investment decision process.

### 2.2 Multimodal financial datasets and vision–language benchmarks

To bridge the gap between textual financial QA and the rich visual environment of practical investing, several multimodal or vision–language datasets have been proposed. MME-Finance (Gan et al., 2024) extends this landscape by introducing a relatively small bilingual multimodal dataset with open-ended questions over financial images, while FAMMA (Xue et al., 2025) focuses on multilingual multimodal QA, including French and English text over financial documents. Fin-Fact (Rangapur et al., 2024) contributes a multimodal fact-checking benchmark that combines textual claims with evidence from financial images, and PDF-VQA (Ding et al., 2023) targets visual question answering over noisy, real-world PDF documents. Sujet-Finance-QA-Vision-100k (Sujet and Allaa Boutaleb, 2024) scales document VQA to 100k financial samples, and FinVis-GPT (Wang et al., 2023) introduces a multimodal LLM specifically for financial chart analysis.

These studies demonstrate that multimodal financial reasoning is both technically feasible and practically valuable. However, when examined from the perspective of interpretable financial reasoning, existing resources remain clearly insufficient. First, most datasets cover only a single category of visual objects (e.g., charts or PDFs), making it difficult to jointly model heterogeneous information sources such as contracts, real-world financial scenes, and candlestick charts within a unified framework. Second, they generally lack explicit human-annotated chain of thought reasoning, which prevents direct training and evaluation of CoT models that align textual reasoning with visual evidence. Third, existing task formulations rarely target the full investment decision making process—such as deriving step-by-step investment conclusions from contract terms or market scenes—ultimately leading to actionable trading or allocation recommendations. Consequently, these datasets can support only isolated capabilities of multimodal robo-advisors but fall short of enabling an end-to-end, interpretable multimodal decision-making system.

### 2.3 Chain-of-thought and multimodal reasoning

In the broader AI literature, chain-of-thought prompting has emerged as a simple yet powerful technique for improving the reasoning capabilities of LLMs. Wei et al. (2023) show that providing a small number of demonstrations with explicit intermediate reasoning steps can dramatically enhance performance on arithmetic, commonsense, and symbolic reasoning benchmarks. Subsequently, a growing body of research (Chellappa et al., 2024; Shao et al., 2024; Hegde et al., 2025; Leong et al., 2024) has incorporated CoT reasoning into multimodal settings.

However, existing multimodal CoT datasets are domain-general and do not capture the specific semantics and constraints of financial decision-making. There is, to the best of our knowledge, no publicly available dataset that combines real-world financial images (contracts, market scenes, and candlestick charts) with high-quality, human-verified chain of thought annotations specifically tailored to investment and advisory tasks.

### 2.4 Identified research gaps and FinErva

Synthesizing the above literature, this study contributes to both the AI and finance domains. First, compared with existing text-only financial QA benchmarks and multimodal datasets, FinErva is the first dataset that simultaneously covers financial contract understanding, real-world financial scene interpretation, and candlestick-based technical analysis, while providing multimodal chain of thought annotations within a unified framework. Second, in contrast to general purpose multimodal CoT benchmarks, FinErva's tasks and annotations are specifically designed for financial decision scenarios, embedding concepts, such as order types, corporate actions, and chart patterns—directly into the reasoning chains.

Third, building on this dataset, this paper proposes a lightweight fine-tuning pipeline for vision–language models with fewer than 0.8 billion parameters, showing that such compact models can achieve expert-level performance on financial multimodal reasoning tasks when trained with appropriate CoT supervision. This direction aligns closely with the development of scalable, interpretable, and financial AI tools, and it directly addresses the practical constraints, such as computational cost, latency, and governance, that large-scale black-box models face when being deployed in production-level robo-advisory systems.

### 2.5 Financial significance and relevance

First, this paper contributes to the field of intelligent financial advisory not only by enhancing interpretability but also by improving the understanding of contracts/disclosures and technical analysis through candlestick charts, thereby optimizing the quality of investment advice and investor outcomes. Currently, a significant challenge faced by intelligent financial advisory systems is how to extract meaningful signals from vast amounts of financial data while providing personalized and compliant recommendations. By incorporating contract and disclosure understanding, this study helps investors identify potential legal risks, such as mis-selling or suitability violations, which is crucial for reducing losses due to information asymmetry or misleading information. Specifically, accurate comprehension of contractual terms and disclosure contents ensures that investment advice aligns with suitability standards, preventing legal disputes and safeguarding investor rights due to improper recommendations. Additionally, technical analysis based on K-line charts provides timely market trend signals, which are vital for risk management and portfolio construction, assisting investors in developing rational trading strategies and reducing emotion-driven investment decisions. The improvements in these two tasks fundamentally enhance the quality of investment advice from intelligent financial advisors, making it not only responsive to market demand but also protective of investors' long-term interests.

More generally, the multimodal chain of thought framework proposed in this study is closely related to financial theory, particularly regulatory requirements such as fiduciary duty, suitability standards, and disclosure obligations in financial services. During the development of intelligent financial advisory systems, regulatory bodies require financial service providers to adhere to fiduciary responsibilities and suitability standards, meaning personalized investment advice must be based on clients' risk tolerance, investment goals, and financial status. This standard mandates that intelligent advisory systems explicitly explain their decision-making process when formulating investment advice. The framework presented in this paper enhances the interpretability of intelligent advisory systems, allowing each piece of investment advice to be traced back to specific contract terms, market data, and technical signals, thus helping financial institutions comply with regulatory requirements and ensure investor rights are protected. On this basis, the research also offers new perspectives for financial regulation. By providing auditable reasoning chains, regulatory bodies can more transparently assess and supervise the decision-making processes of intelligent advisors, ensuring compliance with industry standards and supporting the development of future regulatory frameworks in the financial sector.

Also, this study not only provides contributions to the innovation of financial technologies in terms of data and methodologies but also defines the primary target users of the framework and dataset: intelligent financial advisory developers, academic researchers, and regulatory bodies. For intelligent financial advisory developers, the multimodal chain of thought framework and dataset constructed in this study provide new tools and methodologies, enabling them to deliver more personalized and compliant investment advice in complex financial data environments. For academic researchers, this study offers new data sources and research platforms for further optimization of intelligent advisory systems and financial decision-making research. Specifically, in areas such as risk management in intelligent advisory systems, investor behavior analysis, and compliance review, the application of the FinErva dataset will foster the interdisciplinary integration of finance and artificial intelligence, driving deeper academic exploration. For regulatory bodies, as financial technology evolves rapidly, ensuring that these technologies comply with regulatory requirements and protect investor interests has become an important issue. The framework and dataset provided by this study will assist regulatory bodies in the supervision and assessment of intelligent advisory systems, particularly in ensuring transparency, interpretability, and compliance of investment recommendations, thereby providing a theoretical foundation and practical tools for sustainable development and financial technology regulation in the financial sector.

## 3 The FinErva dataset

### 3.1 Overview

**FinErva** is a multimodal financial question answering dataset designed to facilitate research on chain of thought reasoning based on both visual and textual modalities. It comprises **7.54K multimodal samples**, each data accompanied by: a real-world financial image (e.g., financial contracts, financial statements, candlestick charts, etc.); one correct answer and two distractors crafted to mislead large language models; a detailed caption describing the image content; and a chain-of-thought rationale for solving the corresponding question.

Table 3 demonstrates the split statistics of FinErva. FinErva consists of 7,544 samples divided into two dimensions: FinErva-Pact and FinErva-Price, each with corresponding training, validation, and test splits. FinErva-Pact contains 5,488 samples and is composed of real financial contract QA pairs, focusing on understanding and computation based on textual content within financial documents. FinErva-Price includes 2,056 samples, consisting of real financial candlestick chart QA pairs, targeting detailed interpretation and computation involving complex graphical patterns and quantitative reasoning over complex visual patterns, such as Stock market price analysis.

**Table 3:** FinErva statistics.

**Dataset**	**Split**	**Count**	**Notes**
FinErva-Pact	Train	3,841	Financial contract
	Test	823	
	Val	824	
	Total	5,488	
FinErva-Price	Train	1,440	Financial chart
	Test	308	
	Val	308	
	Total	2,056	
**Overall total**		**7,544**	

### 3.2 Annotation procedure

The annotation process is carefully designed by the author and executed by a professional annotation team. A standard annotation workflow is adopted, consisting of model-assisted pre-annotation followed by human verification. The author confirm that all AI-assisted tools used in this stage are fully legitimate and fully compliant with academic ethical standards.

#### 3.2.1 API annotation

First, we use OpenAI's API to generate two distractor choices, a query, and a chain-of-thought solution for each sample. We employ prompt templates tailored for ChatGPT-o4-mini-high, a model particularly strong in visual–textual reasoning. The complete prompt templates are provided in Appendix B. To facilitate consistent task execution by large language models, the prompts explicitly instruct the model to always assign the correct answer to option A.

Subsequently, during post-processing, the answer options are randomly shuffled to ensure that the correct answers are evenly distributed among the three choices. Importantly, each sample is subsequently reviewed and verified through careful human annotation stage to ensure correctness.

#### 3.2.2 Human annotation

Human annotation constitutes the core of our experiments and has been meticulously designed. Each annotator holds a Master's degree or higher and possesses a proficient level of English (annotators for whom English is not a native language have passed the university English proficiency test in their respective regions). They also have at least three years of professional knowledge in finance, including an understanding of financial contracts and candlestick charts, to ensure that every annotator has the necessary expertise. Each instance in the dataset was independently judged by two human annotators, so that every data point underwent two separate rounds of evaluation. Annotators were fairly paid by 28USD per 200 samples, and their participation was voluntary and conducted under responsible data using guidelines.

The specific guidelines are as follows: Annotators evaluate each data point across four dimensions, from top to bottom, as shown in the Table 4, Problem not valid, No valid option exists, Provided wrong answer, and Reasoning integrity. If an annotator believes that none of the first three dimensions (Problem not valid, No valid option exists, Provided wrong answer) contain errors, meaning all fields in the data do not show obvious mistakes, the annotator will score the reasoning chain (the evaluation of the Reasoning integrity dimension). The score ranges from 5 to 1, representing a spectrum from perfect (5 points) to unacceptable (1 point). If the score given by the annotator is less than 3, it is considered that the evaluation of the fourth dimension (Reasoning integrity) fails, meaning the reasoning chain, although free from obvious errors, does not fully simulate human thought processes. Each data is independently annotated by two annotators, and the data is considered annotated only when both annotators assign a score greater than 3 to the reasoning chain.

**Table 4:** Human-checked error dimension.

**Error dimension**	**Proportion**
Problem not valid	43.72%
No valid option exists	12.28%
Provided wrong answer	18.89%
Reasoning integrity	9.65%
No obvious errors	15.46%

Specifically, when annotators identify a clear error in the data, they will mark the erroneous dimension and provide a reasonable correction, including a correct question, answer, distractions and reasoning. This data will then be returned to the unannotated data queue to be reviewed by another annotator. If the corrected data is deemed no error, it will be sent to a third annotator for verification, and the data will be considered finalized only when at least two annotators approve it.

In practice, after correction by one annotator, the data should not contain obvious errors. A very small number of data may be contentious and will be discussed by the entire annotation team. Another common situation of inconsistency arises when one annotator does not find an obvious error and assigns a reasoning chain score, while another annotator believes there is an error, directly corrects it, and returns it to the unannotated queue. In such cases, the data must be approved by two additional annotators before it is considered finalized. While the process may seem complex, it is easy to implement in practice, as it only requires identifying data points approved by a single annotator and having them annotated by two others. It is important to note that the process of scoring the reasoning chain is highly subjective and cannot be constrained by visualizable rules. Therefore, annotation is considered complete when both annotators agree that the reasoning chain is complete.

Overall, this annotation guideline is entirely human-driven and is strictly enforced.

#### 3.2.3 Data quality assessment

To verify the quality of the constructed dataset, multiple volunteers are recruited to participate in a human evaluation study. The volunteers are divided into two groups: finance majors and random participants, all of whom are graduate students. Samples are randomly drawn from the test set in Price and Pact, presented to the volunteers for manual answering. The results are summarized in Table 2. As shown in the table, the fine-tuned model significantly outperforms random participants and achieves comparable accuracy to that of finance professionals. A comprehensive quantitative evaluation of model accuracy is provided in Section 5. Although the results are subject to potential variance due to the small-sample randomness, they overall demonstrate both the domain expertise embodied in our dataset and the effectiveness of the fine-tuned model. One image is equipped with multiple questions ranging from different aspect. The training data and test data are disjointly split to ensure that no image or question in training and test set both, which prevents data leakage and enables a more reliable evaluation of model performance.

**Figure 1:**
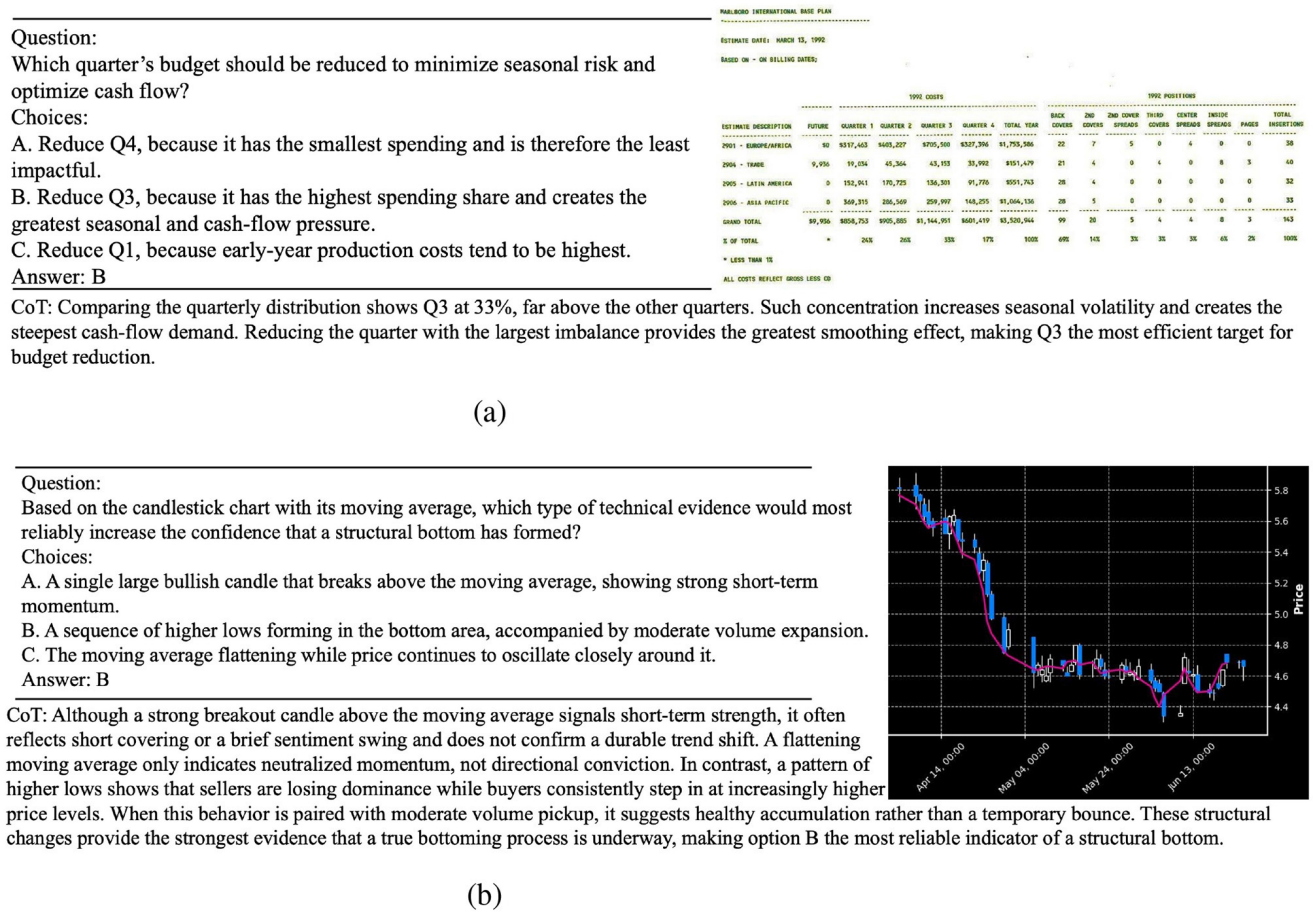
FinErva data. **(a)** A sample in FinErva-pact. **(b)** A sample in FinErva-price.

For better interpretability, we also visualize several representative examples from the dataset in Figure 1, which illustrate the typical difficulty level and reasoning characteristics of our data samples.

### 3.3 Data analysis

#### 3.3.1 Dataset source

FinErva comes from publicly available financial disclosure documents (such as financial reports, company announcements, stock market analysis reports, etc.), financial data service platforms, and historical K-line data from the financial markets (Sujet and Allaa Boutaleb, 2024; Wang et al., 2023)(random selected). All data is publicly sourced and does not involve any confidential or restricted data sources. Specifically, the dataset includes data from different markets, industries, and asset classes, ensuring the dataset's breadth and representativeness.

#### 3.3.2 Data structure

Figure 1 demonstrates one sample in FinErva-Pact and one sample in FinErva-Price. All questions in the dataset are formatted as multiple-choice, each with a single correct answer. Every question is deliberately constructed to require complete reliance on the visual input, ensuring that the model truly utilizes multimodal information. Additionally, each question is accompanied by a manually verified chain of thought rationale to guide model learning. The questions span various forms, including text understanding within images, numerical reasoning based on visual content, and analysis of real-world financial images. The overall goal is to equip models with strong visual reasoning capabilities in the financial domain. A complete example of the dataset is provided in the Appendix A. At the same time, we ensure the legality of all the data and guarantee that no sensitive or confidential information is involved and also ask readers to ensure under legal restrictions of their respective regions when using this dataset.

#### 3.3.3 Data distribution

FinErva spans a comprehensive and diverse set of question types with varying levels of complexity, reflecting a typical retail investment advisory scenario. The Pact subset consists of simple question-answering tasks, whereas the Price subset focuses on complex understanding-reasoning tasks. Table 3 shows detail size of FinErva. Tables 5, 6 report the distribution of question types across the two subsets, and we intentionally balance the number of questions in each category, ensuring that the capabilities learned by the model are comprehensive rather than biased toward any single modality or task. Training, test, validation sets are keeping the distribution.

**Table 5:** Distribution of question types in the pact subset.

**Question type**	**Proportion (%)**
Numerical reasoning	31.82
Textual comprehension	37.15
Information retrieval	31.03

**Table 6:** Distribution of question types in the price subset.

**Question type**	**Proportion (%)**
Basic information comprehension	23.01
Basic numerical reasoning	21.98
Advanced computations	19.99
Technical-analysis tactics	19.02
Chart patterns	16.00

Specifically, the Pact subset covers comprehensive question–answering scenarios involving financial contracts. The Price subset encompasses nearly all complex candlestick-chart QA scenarios, including: (i) basic information comprehension: opening price, closing price, and intraday range; (ii) basic numerical reasoning: moving averages, relative volume (volume ratio), volatility, and percentage return; (iii) advanced computations: MACD, RSI, Williams %R (WR), and actual traded volume; (iv) technical analysis tactics: golden cross, death cross, and WR overbought/oversold signals; and (v) chart patterns: three black crows, ascending channel, descending channel, and the “air-refueling” (mid-trend consolidation) pattern.

## 4 Methodology

**Figure 2:**
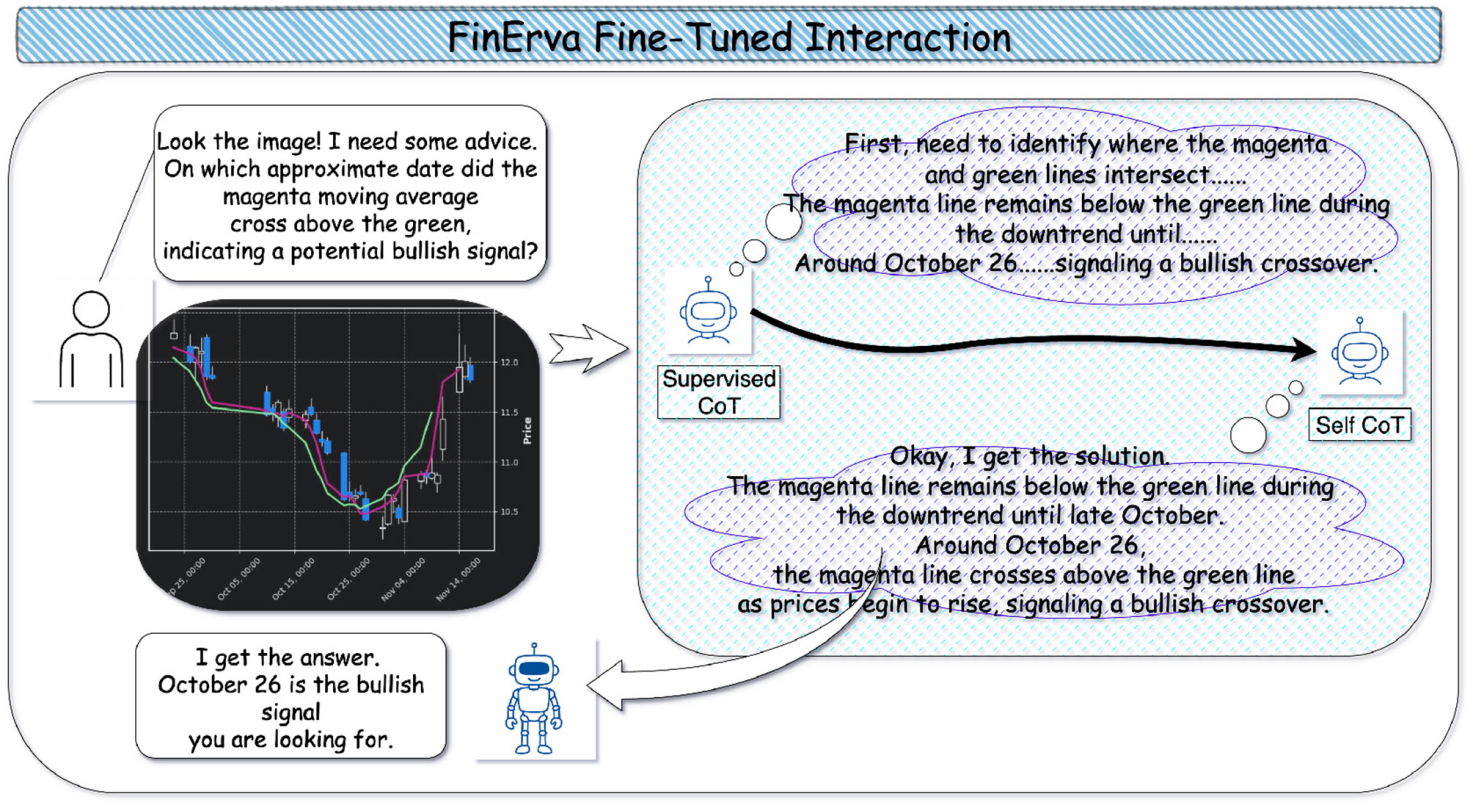
The pipeline for FinErva fine-tuned model.

This section describes the training procedure, which is shown in Figure 2. In this work we adopt a chain of thought paradigm because financial decision-making typically involves multi-step reasoning rather than one-shot classification. Supervising the model to generate intermediate rationales encourages it to decompose each task into economically meaningful steps instead of relying on shallow correlations. Building on this idea, the two stages framework first uses Supervised-CoT-Learning to imprint domain-faithful reasoning patterns from human annotated chains, and then applies Self-CoT-Learning to expand and stabilize this behavior on a larger set of examples without additional expert labeling. This design not only yields higher predictive accuracy in experiments, but also produces explanations that can be inspected by practitioners and regulators, making the resulting system better aligned with the transparency and accountability requirements of robo-advisory and risk management.

### 4.1 Task definition

From a financial decision-support perspective, each instance in FinErva is designed to mimic a concrete advisory or analysis step, such as interpreting a contract clause, assessing a disclosure, or reading a candlestick pattern before making a trading or allocation decision. Formally, let I denote the space of visual inputs (e.g., M×N RGB images of contracts, screenshots, or candlestick charts), Q the discrete sequence space of natural-language questions posed in a financial context, R the space of chain of thought reasoning sequences, and A the space of answer sequences (multiple choice decisions).

A single sample in the multimodal financial question answering task can thus be formalized as a quadruple:


$$\begin{array}{l} x=\left(x_{\text{vision}},x_{\text{lang}},r^{⋆},a^{⋆}\right)\in I\times Q\times R\times A \end{array}$$ (1)

where *x*_vision_ is the visual input (such as a candlestick chart or contractual page), *x*_lang_ is the corresponding financial question (e.g., about fees, risk, or price behavior), *r*^⋆^ is an expert-style intermediate reasoning process that articulates economically and legally meaningful steps, and *a*^⋆^ is the final decision or answer. Intuitively, (*r*^⋆^, *a*^⋆^) plays the role of a “transparent advisory decision,” making explicit how an informed analyst would move from raw information to an actionable conclusion.

### 4.2 Target mapping

The goal is to learn a parameterized model *F*_θ_ that takes the two modalities as input and outputs both a reasoning chain and a final decision:


$$\begin{array}{l} F_{\theta }:\left(I,Q\right)\to R\times A \end{array}$$ (2)

In financial terms, *F*_θ_ can be viewed as an approximate decision policy that maps a given disclosure or market snapshot together with a user query into an interpretable rationale and a recommendation. Learning *F*_θ_ therefore aims not only at predicting the correct answer, but also at recovering a step-by-step inference that can be scrutinized by practitioners, risk managers and regulators, in line with transparency and suitability expectations in robo-advisory.

### 4.3 Training target

The annotated dataset is denoted by D, where each training instance consists of a real visual input *x*_vision_, a natural language question *x*_lang_, an expert-annotated reasoning chain *r*^⋆^, and a ground-truth answer *a*^⋆^. The training objective L(θ) is to minimize the negative log-likelihood of the pair (*r*^⋆^, *a*^⋆^):


$$\begin{array}{l} L\left(\theta \right)=E_{\left(x_{\text{vision}},x_{\text{lang}},r^{⋆},a^{⋆}\right)\sim D}\left[-\log p_{\theta }\left(r^{⋆},a^{⋆}|x_{\text{vision}},x_{\text{lang}}\right)\right] \end{array}$$ (3)

where


$$\begin{array}{l} p_{\theta }\left(r,a|x_{\text{vision}},x_{\text{lang}}\right)=\overset{T_{R}}{\underset{t=1}{\prod }}p_{\theta }\left(s_{t}|x_{\text{vision}},x_{\text{lang}},s_{<t}\right) \end{array}$$

$$\begin{array}{ll} \cdot & \overset{T_{A}}{\underset{k=1}{\prod }}p_{\theta }\left(y_{k}|x_{\text{vision}},x_{\text{lang}},r,y_{<k}\right) \end{array}$$ (4)

and Equation 3 is equivalent to maximizing *p*_θ_(*r, a*∣*x*_vision_, *x*_lang_), the joint conditional likelihood of the data. *r* = (*s*_1_, …, *s*_*T*_*R*__) denote the tokenized reasoning sequence with length *T*_*R*_, and *a* = (*y*_1_, …, *y*_*T*_*A*__) denote the tokenized answer sequence with length *T*_*A*_.

Economically, this objective encourages the model to learn not only which answers are correct, but also which *reasoning patterns* are consistent with expert financial practice: for instance, checking key cost and risk disclosures before judging product suitability, or combining trend, volatility and support/resistance levels before characterizing a candlestick configuration. Maximizing the joint likelihood *p*_θ_(*r, a*∣*x*_vision_, *x*_lang_) therefore aligns the model with the dual goal of modern advisory systems: accurate decisions and traceable, domain-consistent justifications.

### 4.4 Two stages optimization

Formulation (Equation 4) reflects an auto-regressive decomposition under the *reasoning-then-answering* paradigm: the model first generates a complete reasoning chain *r* ∈ R^⋆^, conditioned on the input pair (*x*_vision_, *x*_lang_), and subsequently generates the final answer *a* ∈ A^⋆^ based on both the input and the generated reasoning chain.

#### 4.4.1 Vision encoding

Since most lightweight text generation models do not natively support multimodal inputs, visual features must be extracted first by using a Vision Transformer (ViT) (Dosovitskiy et al., 2021; Touvron et al., 2021). Specifically, a visual encoding layer is introduced by removing the classification head of the ViT. The encoded visual features, $x_{\text{vision}}\in I\subset {\mathbb{R}}^{H\times W\times 3}$, are then directly concatenated with the text embeddings and fed into the model during training, which significantly reduces overall training time.

#### 4.4.2 Supervised-CoT-learning

In this stage, the training target is, given the input pair (*x*_vision_, *x*_lang_), to minimize:


$$\begin{array}{l} L_{\text{CoT}}=-\overset{T_{R}}{\underset{t=1}{\sum }}\log p_{\theta }\left(s_{t}|x_{\text{vision}},x_{\text{lang}},s_{<t}\right) \end{array}$$ (5)

which denotes the model first generating a sequence of reasoning steps *s*_1_, *s*_2_, …, *s*_*T*_*R*__, conditioned on the given image and question. Each step *s*_*t*_ is generated based on the previously generated steps *s*_<*t*_. This encourages the model to reproduce the expert-annotated step-by-step reasoning sequence *r*^⋆^.

#### 4.4.3 Self-CoT-learning

In the second stage, once the model has generated its own reasoning chain $\hat{r}$, we concatenate this chain with the original image–question pair and use the resulting triplet as the input to this stage:


$${\tilde{x}}_{\text{lang}}=\left[x_{\text{lang}}∥\hat{r}\right],$$

and the training target is to minimize:


$$\begin{array}{l} L_{\text{Ans}}=-\overset{T_{A}}{\underset{k=1}{\sum }}\log p_{\theta }\left(y_{k}|x_{\text{vision}},{\tilde{x}}_{\text{lang}},y_{<k}\right) \end{array}$$ (6)

which denotes the model generating the answer tokens *y*_*k*_ after the complete reasoning chain *r*, conditioned on the inputs (*x*_vision_, *x*_lang_, *r*) and the previously generated answer tokens *y*_<*k*_. This encourages the model to generate more refined and accurate answers conditioned on its self-generated reasoning.

## 5 Experiments

### 5.1 Training details

#### 5.1.1 Lightweight models

This work focuses on training lightweight models, as their lower deployment cost offers significant advantages for future applications in personalized intelligent financial advisory systems. All models used in the experiments have fewer than 0.8 billion parameters, with the smallest model containing only 0.06 billion parameters, making it feasible to train on a single NVIDIA RTX 3090 GPU.

#### 5.1.2 Training time

To further reduce training time, all experiments are conducted using two RTX 3090 GPUs in parallel, with each training run completed in under 8 hours. Small models only using about 2 hours. These statistics are provided solely to illustrate that the experiments adhere to the study's objective of maintaining low computational cost. They are based only on the local experimental environment and are intended for reference rather than for statistical inference.

#### 5.1.3 Cross-validation

To prevent any potential data leakage among the training, validation and test portions of the 0.70/0.15/0.15 split, all reported performance metrics are obtained by averaging over five-fold cross-validation conducted exclusively within the combined 85% (train and val) non-test subset of the data. After selecting the optimal hyperparameters via cross-validation, the model is retrained on the entire 85% training pool and subsequently evaluated once on the strictly held-out 15% test set to obtain the final reported results.

#### 5.1.4 Hyperparameter selection

To guarantee experimental reproducibility, we set a global random seed at 42. For the remaining hyperparameter choices, the settings may vary across different training devices and environments. Taking batch size as an example, an excessively large batch size may lead to out-of-memory issues, whereas an overly small batch size may result in under utilization of computational resources. The appropriate choice depends on the specific experimental setup. The selection of hyperparameter can cause slight fluctuations in training results, which is widely acknowledged in practice.

### 5.2 Evaluation

All models' performance will be assessed from two complementary perspectives in three different training stages, which are reported in Table 7 respectively. First, zero-shot evaluation on test set; second, in the two-stage chain of thought pipeline without fine-tuning; third, after fine-tuning on FinErva dataset.

**Table 7:** FinErva-Pact results.

	**Google-flan-T5-small**	**Google-flan-T5-base**	**Google-flan-T5-large**	**Lamini-flan-T5-77M**	**Lamini-flan-T5-248M**	**Lamini-flan-T5-783M**	**Flan-alpaca-base**	**Flan-alpaca-large**
Parameters	60M	220M	770M	77M	248M	783M	220M	770M
Accuracy/%	20.77	20.59	20.59	20.59	20.59	20.59	20.59	20.59
ROUGE-1/%	33.61	38.95	38.30	33.94	43.65	45.64	38.81	44.07
ROUGE-2/%	11.40	14.47	15.29	11.15	16.62	17.99	15.15	17.15
ROUGE-L/%	27.97	30.41	30.86	26.63	33.42	34.97	30.48	34.29
Similarity/%	59.33	66.35	67.01	59.46	70.32	72.03	67.36	71.69

Each evaluation has three lines, which indicates models' performance under three different evaluation stages: first, zero-shot evaluation on test set; second, in the two-stage chain of thought pipeline without fine-tuning; third, after fine-tuning on FinErva dataset. The red color values indicate the best performance model.

#### 5.2.1 Accuracy

Because each question has exactly one correct choice, Accuracy directly reflects decision reliability. T denotes the test space, ${â}_{i}\in A$ the predicted option for sample *i*, and $a_{i}^{⋆}$ the ground truth. Accuracy is calculated as following:


$$\begin{array}{l} Accuracy\left(Acc\right)=\frac{1}{\left|T\right|}\overset{\left|T\right|}{\underset{i=1}{\sum }}1\left[{â}_{i}=a_{i}^{⋆}\right]. \end{array}$$ (7)

#### 5.2.2 ROUGE

Using ROUGE (Recall-Oriented Understudy for Gisting Evaluation) (Lin, 2004) score to quantify the solution $\hat{r}$ generated by model under given *r*^⋆^, and computing Recall and Precision:


$$\begin{array}{l} R=\frac{\underset{g\in r^{⋆}}{\sum }\min\left\{{\text{Count}}_{\hat{r}}\left(g\right),{\text{Count}}_{r^{⋆}}\left(g\right)\right\}}{\underset{g\in r^{⋆}}{\sum }{\text{Count}}_{r^{⋆}}\left(g\right)}, \end{array}$$

$$\begin{array}{l} P=\frac{\underset{g\in r^{⋆}}{\sum }\min\left\{{\text{Count}}_{\hat{r}}\left(g\right),{\text{Count}}_{r^{⋆}}\left(g\right)\right\}}{\underset{g\in \hat{r}}{\sum }{\text{Count}}_{\hat{r}}\left(g\right)} \end{array}$$ (8)

where *g* spans all reference *N*-grams (with *N*∈{1, 2}), and ROUGE-L is computed via the longest common subsequence (LCS). **ROUGE-1**, **ROUGE-2**, and **ROUGE-L** are reported in results. Tables 7, 8 report the harmonic mean of recall (*R*) and precision (*P*), computed as


$$F_{1}=\frac{2\cdot R\cdot P}{R+P}.$$

**Table 8:** FinErva-Price results.

	**Google-flan-T5-small**	**Google-flan-T5-base**	**Google-flan-T5-large**	**Lamini-flan-T5-77M**	**Lamini-flan-T5-248M**	**Lamini-flan-T5-783M**	**Flan-alpaca-base**	**Flan-alpaca-large**
Parameters	60M	220M	770M	77M	248M	783M	220M	770M
Accuracy/%	22.61	18.88	18.88	22.61	22.61	18.88	18.88	18.88
ROUGE-1/%	12.24	24.32	32.56	10.85	23.38	26.90	26.31	32.56
ROUGE-2/%	1.04	8.22	11.33	1.74	7.19	9.81	8.74	11.33
ROUGE-L/%	10.92	20.25	24.58	9.22	16.96	19.91	20.31	24.58
Similarity/%	23.54	53.10	62.83	27.40	57.52	60.32	56.53	62.83

Each evaluation has three lines, which indicates models' performance under three different evaluation stages: first, zero-shot evaluation on test set; second, in the two-stage chain of thought pipeline without fine-tuning; third, after fine-tuning on FinErva dataset. The red color values indicate the best performance model.

#### 5.2.3 Similarity

The similarity score is obtained by first encoding both the generated and the annotated chains of thought into embeddings (as **e**_*a*_, **e**_*b*_) using the Sentence-BERT (Reimers and Gurevych, 2019), and then computing their cosine similarity. In experiments, we adopt the lightweight all-MiniLM-L6-v2 Sentence-BERT model, which is specifically designed for sentence embedding and semantic similarity computation. This choice ensures the reliability of the experimental results while maintaining the lightweight nature of our approach. The computation is given by:


$$\begin{array}{l} {\mathrm{sim}}_{\cos}\left({\text{e}}_{a},{\text{e}}_{b}\right)=\frac{{\text{e}}_{a}^{⊤}{\text{e}}_{b}}{∥{\text{e}}_{a}{∥}_{2}∥{\text{e}}_{b}{∥}_{2}} \end{array}$$ (9)

This metric reflects the semantic relatedness between the generated chain of thought and the human-annotated chain of thought.

### 5.3 Results and analysis

In the financial domain, the output modality is almost exclusively textual, and the input modality is also predominantly either text-only or text–vision. Accordingly, the author fine-tuned the classical text-to-text T5 (Text-to-Text Transfer Transformer) models, which have robust performance on text-vision2text task (Raffel et al., 2020; Wei et al., 2021). All models selected in experiments are lightweight, and results demonstrate that these lightweight models still exhibit strong capabilities in financial question answering tasks. The evaluated models include the google/Flan-T5 family (Chung et al., 2022), as well as instruction-tuned variants such as the LaMini-Flan-T5 family (Wu et al., 2023) and the alpaca-flan family (Bhardwaj and Poria, 2023).

#### 5.3.1 Main results

The test results of the eight evaluated models on the two subsets are presented in Tables 7, 8. In the zero-shot evaluation, all models achieve comparable accuracy on both subsets. However, in the two-stage evaluation without fine-tuning, accuracy improves significantly across all models, demonstrating the pronounced effect of chain-of-thought reasoning in multimodal financial QA tasks. Overall, there is a positive correlation between model size and accuracy, which is consistent with expectations. From a financial perspective, moving from low performance to substantially higher accuracy on FinErva-Pact translates into a lower probability that an automated system misinterprets key contractual or disclosure items, thereby reducing the risk of mis-selling and suitability breaches. Similarly, the gains observed on FinErva-Price indicate that the models more reliably recognize economically meaningful price configurations and basic risk signals in candlestick charts, which is a prerequisite for supporting trading discipline and avoiding systematically biased entry or exit decisions. Although experiments are conducted in an offline setting, these improvements in predictive accuracy can be interpreted as proxies for fewer interpretive errors and enhanced investor protection when such components are embedded into real-world robo-advisory workflows.

#### 5.3.2 Higher learning cost in larger models

It is worth noting that in part of the FinErva-Price results, large-parameter models exhibit slightly lower answer accuracy after generating reasoning chains compared to medium-sized models (as shown in the second row of the Accuracy metric). A closer inspection of the intermediate reasoning chains reveals that the semantic richness and diversity of the CoTs generated by medium-sized models are noticeably higher than those of the large models.

This phenomenon can be explained by two main factors. First, the reasoning ability of different model series—each fine-tuned under distinct instruction sets—naturally varies in financial QA tasks. This is reflected in the first row of the Accuracy metric and is primarily determined by the pretraining stage, rather than the fine-tuning process or the dataset itself.

**Figure 3:**
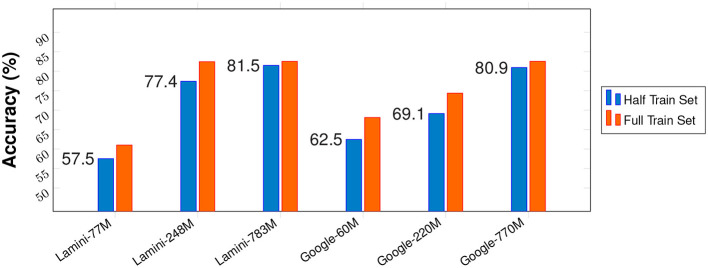
Comparison of model performance under different training set scales.

Second, larger models require significantly higher fine-tuning costs and larger training datasets. In the early stages of fine-tuning (the first iteration, as shown in the second accuracy row), large models demand a greater number of samples to achieve stable learning. As a result, they may initially underperform compared to medium-sized models. However, large models typically learn faster, and once the data scale reaches a sufficient threshold, they often enter a performance plateau, where further improvements become marginal. As shown in Figure 3, when trained with only 50% of the full dataset, both small and medium models experience a noticeable drop in performance, whereas the large model's accuracy remains almost unchanged. This indicates that the small and medium models are still in the active learning phase, while the large model has already reached its saturation or plateau stage.

Such behavior is particularly evident in more complex reasoning tasks. In contrast, this phenomenon does not appear in FinErva-Pact, further confirming the high quality and stability of our dataset.

#### 5.3.3 Limitations of ROUGE for CoT evaluation

Although the ROUGE metric is employed in this work to evaluate the semantic similarity of reasoning chains, the observed improvements in ROUGE scores before and after fine-tuning are relatively limited. This indicates the inherent limitations of ROUGE when applied to reasoning-based or semantically rich tasks. As a lexical overlap–based metric, ROUGE primarily focuses on surface-level token or phrase matching, without genuinely capturing the semantic meaning or logical structure of the reasoning process. Moreover, the metric is highly sensitive to the annotators' linguistic and cognitive styles, which may differ substantially from the instructional patterns learned by large pre-trained models. Therefore, while ROUGE can provide a coarse quantitative reference, it does not fully reflect the semantic or reasoning-level alignment between the model-generated and human-annotated CoTs.

#### 5.3.4 Initial accuracy lower than random-guessing

As results in the first line of Accuracy, the fact that the initial accuracy falls below the random-guessing (0.33) baseline itself attests to the intrinsic complexity of this dataset. Because the questions incorporate substantial visual information, the model's predictions are easily misled, preventing it from extracting the correct answer from such rich visual content. Indeed, an initial accuracy lower than the mathematical expectation of random selection precisely indicates that the model is striving to interpret both complex visual and textual signals—and in doing so, it further highlights the dataset's challenging nature.

#### 5.3.5 Not perfect similarity score

Similarity measure precisely captures the semantic relationship between the generated and annotated chains of thought. Results in Tables 7, 8 demonstrate that the chains learned by the large model are semantically similar to the human-annotated chains, even though the numeric similarity scores are not particularly high. This is because cosine similarity reflects only the structural resemblance between two texts and does not truly capture their semantic content. To further illustrate this phenomenon, we conducted an additional experiment using the following three sentences:

sent-A = “I attended a meeting at the office this morning.”

sent-B = “I just wrapped up an early-morning business discussion.”

sent-C = “I had breakfast at the office this morning.”

The computed semantic similarity by all-MiniLM-L6-v2 between A and B is only 0.47, despite the two sentences expressing nearly identical meanings. In contrast, the similarity between A and C reaches 0.63, even though their semantic content is entirely unrelated. This suggests that current Sentence-BERT predominantly capture surface-level lexical or syntactic resemblance rather than genuine semantic equivalence. Consequently, quantitative semantic similarity metrics exhibit inherent limitations, which explains why the similarity evaluation indicators in our experiments are not perfectly aligned with true semantic consistency.

#### 5.3.6 Thinking inertia

Interestingly, an experiment shows that when the correct answer is always placed at option A, model tends to learn this latent pattern. On the test set, this setup leads to a 1–2 percentage point increase in accuracy compared to the shuffled version. It means that, if all correct answers are always presented in the same position, the large model may learn this superficial pattern. However, as the experimental results reveal, the impact of this positional bias is negligible.

## 6 Conclusion

This paper proposes FinErva as a new building block for data-driven, yet interpretable, financial decision support. From a financial perspective, the framework responds to three structural needs that arise in modern investment practice: (1) the ability to reason jointly over heterogeneous information sources such as contracts, disclosures, market scenes and candlestick charts; (2) the requirement that automated advice be transparent enough to withstand scrutiny from investors, risk managers and regulators; and (3) the necessity of keeping modeling and deployment costs at a level that is feasible for financial institutions beyond a small set of frontier AI labs.

FinErva is, to our knowledge, the first multimodal chain-of-thought dataset specifically designed for financial reasoning. It integrates real-world financial contracts and candlestick charts with fine-grained reasoning annotations, yielding 7,544 manually validated samples across two complementary subsets: FinErva-Pact for contract and disclosure understanding, and FinErva-Price for price-pattern and technical-analysis reasoning. Each instance includes multimodal inputs and a human-supervised reasoning chain, which enables joint evaluation of answer accuracy and the quality of the underlying rationale rather than focusing solely on black-box predictive performance.

On top of this dataset, we design and empirically validate a two-stage training paradigm: Supervised-CoT Learning followed by Self-CoT Refinement, for lightweight vision–language models with fewer than 0.8 billion parameters. The results show that explicit reasoning supervision substantially improves performance over zero-shot and standard fine-tuning baselines and allows lightweight models to approach the reasoning competence of domain experts while being trainable on commodity hardware. For practitioners, this suggests that expert-level multimodal reasoning for tasks such as contract review, chart-based signal extraction and scenario analysis does not necessarily require frontier models, but can be achieved through targeted, domain aligned CoT adaptation.

Beyond serving as a standalone framework, FinErva highlights the broader role of structured reasoning supervision in the design of trustworthy financial AI. The findings indicate that the path toward robust multimodal robo-advisory and risk-management systems lies not only in scaling models, but also in aligning their intermediate reasoning processes with the interpret ability and audit ability requirements of financial economics.

This work has several limitations that open avenues for future research. The current dataset focuses on English-language materials and a subset of visual artifacts (contracts and candlestick charts); extending FinErva to tabular and time-series data, additional document types and multilingual, cross-market settings would strengthen its coverage of global financial practice. From an ethical perspective, the dataset may contain biases, face limitations in its generalizability across jurisdictions and market structures, and raise questions about how to enhance decision-making capabilities while simultaneously addressing ethical and governance concerns. Moreover, integrating FinErva into open evaluation frameworks for retrieval-augmented and reinforcement-tuned reasoning systems would facilitate systematic comparison of alternative architectures for interpretable financial AI. Taken together, these directions position FinErva as a foundation for the next generation of personalized, explainable and operationally viable multimodal intelligence in finance.

## Data Availability

The source code of the project is available at [https://github.com/JerryChi222/FinErva-Interpretable-Multimodal-Reasoning-for-Robo-Advisory.git], and the dataset can be accessed at [https://huggingface.co/datasets/jerrychi/FinErva].

## Footnotes

1 Minerva is a goddess of wisdom in Roman mythology.
